# The Impact of Tartrazine on DNA Methylation, Histone Deacetylation, and Genomic Stability in Human Cell Lines

**DOI:** 10.3390/nu17050913

**Published:** 2025-03-06

**Authors:** Afshin Zand, John M. Macharia, Istvan Szabó, Gellért Gerencsér, Ádám Molnár, Bence L. Raposa, Timea Varjas

**Affiliations:** 1 Department of Public Health Medicine, Medical School, University of Pécs, 7624 Pécs, Hungary; bokor28@yahoo.co.uk (I.S.); gellert.gerencser@gmail.com (G.G.); timea.varjas@aok.pte.hu (T.V.); 2 Doctoral School of Health Sciences, Faculty of Health Sciences, University of Pécs, 7621 Pécs, Hungary; johnmacharia@rocketmail.com; 3 Preclinical Research Center, Medical School, University of Pécs, 7624 Pécs, Hungary; molnar.adam2@aok.pte.hu; 4 Institute of Basics of Health Sciences, Midwifery and Health Visiting, Faculty of Health Sciences, University of Pécs, 7621 Pécs, Hungary; raposa.laszlo@pte.hu

**Keywords:** tartrazine, HaCaT, HepG2, A549, DNMTs, HDACs, comet assay, quantitative real-time PCR, azo dye, epigenetics, food colorant, gene expression

## Abstract

Background/Objectives: Tartrazine (TRZ), a synthetic red azo dye derived from coal tar, is widely used as a food colorant in various food products, pharmaceuticals, and cosmetics. This study aims to investigate the impact of TRZ on the expression levels of DNA methyltransferases (*DNMT1*, *DNMT3a*, and *DNMT3b*) and histone deacetylases (*HDAC5* and *HDAC6*). Additionally, we evaluate genomic DNA stability using the alkaline comet assay in three human cell lines: immortalized human keratinocyte (HaCaT), human hepatocellular carcinoma (HepG2), and human lung adenocarcinoma (A549). The research question focuses on whether TRZ exposure alters epigenetic regulation and DNA integrity, potentially implicating its role in carcinogenesis. Methods: The selected human cell lines were exposed to different concentrations of TRZ (20 µM, 40 µM, and 80 µM), with DMBA serving as a positive control. After treatment, we quantified the expression levels of *DNMT1*, *DNMT3a*, *DNMT3b*, *HDAC5*, and *HDAC6* using quantitative real-time PCR. Additionally, we assessed DNA fragmentation via the alkaline comet assay to determine the extent of DNA damage resulting from TRZ exposure. Results: Our findings indicate that TRZ significantly upregulates the expression of *HDAC5*, *HDAC6*, *DNMT1*, *DNMT3a*, and *DNMT3b* in comparison to the control group. Furthermore, TRZ exposure leads to a notable increase in DNA damage, as evidenced by elevated tail moments across all examined human cell lines. Conclusions: These results suggest that TRZ may play a role in carcinogenesis and epigenetic modifications. The observed upregulation of *DNMTs* and *HDACs*, coupled with increased DNA damage, highlights the potential risks associated with TRZ exposure. Further research is necessary to explore these mechanisms and assess their implications for human health.

## 1. Introduction

Food coloring encompasses dyes, pigments, or compounds that provide color to food, drugs, or cosmetics. These substances are regulated by the Food and Drug Administration (FDA) colorants to ensure their safety. Dyes are classified based on certification requirements. According to FDA and European Food Safety Authority (EFSA) guidelines, these colorants restore lost color and improve the aesthetic appeal of food items [1,2]. The beverage and food sectors employ a lot of colorants, such as synthetic azo dyes and pigments derived from natural sources, to enhance sensory aspects and visual appeal [3]. Due to their appealing coloring qualities, variety of colors, endurance, and affordability, there has been an estimate that over eight million tons of artificial food colorants are made each year [4].

These colorants are often present in commonly consumed foods, typically without the consumer’s awareness, highlighting the need to investigate their biological impacts. Given the established link between nutrition and health and the increasing emphasis on quality of life, many studies have identified dyes that pose health risks, potentially contributing to conditions like childhood hyperactivity, urticaria, asthma, and allergic rhinitis [5].

Azo dyes have drawn significant interest because of their potential health hazards, such as negative consequences on the liver, kidneys, and neurological system [6,7,8], and their role in promoting inflammation [3]. The harmful impact of azo dyes is primarily due to the production of aromatic amines from the breakdown of the aryl-N=N-aryl group by gut microbiota [9]. It is believed that aromatic amines are harmful, carcinogenic, and mutagenic [10].

**Figure 1:**
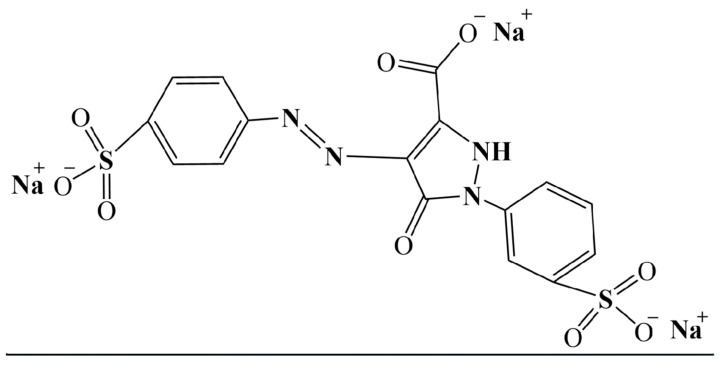
Chemical structure of tartrazine.

One of the most popular food colorants is tartrazine (TRZ, E102) (Figure 1), a synthetic azo dye with a lemon-yellow hue. TRZ is found in various grocery items like soft and sports drinks, jellies, dressings, chewing gums, and non-food products like beauty and pharmaceutical products [11,12]. Moreover, TRZ is also an affordable substitute for saffron in some regions [13]. The EFSA’s 1964 assessment outlined TRZ’s characteristics, purity standards, and toxicological data, establishing an acceptable daily intake (ADI) of 0–7.5 mg/kg body weight. In 2016, the ADI for TRZ was increased to 0–10 mg/kg body weight, due to no observed adverse effect level (NOAEL) of 984 mg, as there is insufficient evidence of adverse effects [14]. However, studies on the health impacts of TRZ have been controversial, with severe concerns, including DNA damage [15], hyperactivity [16], central nervous system alterations [17], and allergic reactions [18,19,20,21].

Numerous studies have examined TRZ’s effects on experimental animals. For example, administering 700 mg/kg of TRZ caused cell growth dysregulation and brain hemorrhage in adult rats without resulting in lipid peroxidation [6]. In our previous research, we discussed TRZ’s histopathological effects on rat liver and kidney tissues, showing vacuolation, swelling, necrosis, and pyknosis. This red-azo dye has the potential to damage DNA in both in vivo and in vitro environments. Research utilizing the comet assay method has shown genotoxic effects in the white blood cells of rats exposed to tartrazine [22]. Both TRZ and its metabolites demonstrate genotoxic effects and cytotoxicity when present in high concentrations. The harmful effects of azo dyes stem from the formation of aromatic amines, which occur due to the cleavage of aryl-N=N-aryl bonds [23]. These byproducts undergo oxidation to N-hydroxy derivatives, leading to redox balance disturbances and neurotoxicity, as indicated by increased levels of malondialdehyde (MDA) and inhibition of the activity of antioxidant enzymes. In one study, Rats given TRZ had higher brain concentrations of acetylcholine (Ach) and gamma-aminobutyric acid (GABA) as well as lower levels of dopamine (DA). TRZ caused oxidative destruction of the liver and kidneys of rats at dosages of 10 and 100 mg/kg [8]. Furthermore, adults who were administered 125–500 mg/kg TRZ for 30 days showed alterations in memory and learning, as well as lowered protective antioxidants [24].

DNA methyltransferases (DNMTs) and histone deacetylases (HDACs) are enzyme groups with significant roles in epigenetics. A class of enzymes called DNMTs adds a methyl group to DNA cytosine residues at position five, which is vital to genomic stability and integrity. DNA methylation regulates gene expression by suppressing specific genes through transcription inhibition, often by methylating gene promoter regions [25]. Three active DNMT members exist: DNMT1, DNMT3A, and DNMT3B. Through abnormal DNA methylation, dysregulation of these enzymes can result in chromosomal instability and cancer. Research indicates that excessive expression of DNMTs may lead to tumor-suppressor gene methylation, silencing them and contributing to cancer [26]. There is a clear correlation between several tumors in humans, such as lung, breast, and colon cancers, and excessive methylation of DNA repair gene promoters [27].

HDACs, tasked with removing acetyl groups from histones, influence the expression of many genes. Research shows HDACs are pivotal in modulating acetylation levels, a phenomenon thoroughly examined in diverse cancer cells. HDACs are vital in initiating, advancing, and promoting carcinogenesis [28]. HDAC2, HDAC3, and HDAC8, part of the HDAC enzyme family, are crucial in controlling cell proliferation. They interact with different co-repressors and transcriptional repressors, which include polycomb group proteins, to inhibit the expression of genes that encourage cell division. Research suggests HDAC genes stimulate angiogenesis, invasion, and migration while suppressing cell cycle inhibitors, distinction, and apoptosis [29,30].

This research explored the relationship between TRZ and the expression levels of *HDAC5*, *HDAC6*, *DNMT1*, *DNMT3a*, and *DNMT3b*. The assessment was carried out using qRT-PCR analysis on various cell lines, including immortalized keratinocyte (HaCaT), lung carcinoma (A549), and human liver cancer (HepG2).

## 2. Materials and Methods

### 2.1. Cell Culture

HaCat: The HaCat human immortalized keratinocyte cell lines (Cytion catalog number 300493, Eppelheim, Germany) particularly derived from adult human skin, these cells demonstrate comparable biological characteristics to normal human keratinocytes, making them an ideal cellular model for investigating dermal toxicity [31]. The HepG2 cell line (ATCC; Manassas, VA, USA) originated from a liver cancer case of a Caucasian suffering from hepatocellular carcinoma. HepG2 cells were chosen for this study due to their retention of morphological and biochemical features like those of regular human hepatocytes, as reported in the literature [32,33]. The A549 cell line (representing human lung cancer) was purchased from the American Type Culture Collection (ATCC; Manassas, VA, USA), which is representative of lung cancer in humans. A549 cells are ideal for research on the biology of cancer [34]. The Dulbecco’s altered Eagle’s medium (DMEM) (Merck-Sigma-Aldrich, Budapest, Hungary, catalog number D6429) was used to cultivate these cells, supplemented with an additional 10% Fetal Bovine Serum (FBS) from Biosera, Cholet, France, and 1× penicillin/streptomycin solution (Thermo Fisher Scientific, Budapest, Hungary catalog number 15140122). Cultures were kept at 37 °C in a 5% CO_2_ atmosphere. Following the guidelines provided by the manufacturer, the designated cell lines were seeded onto 6-well plates. A TC20 automated cell counter (Bio-Rad Inc., Hercules, CA, USA) was used to measure the percentage of seeded cells. The experiment was conducted when reaching 50–60% confluence.

### 2.2. Tartrazine Treatment

Three different concentrations of TRZ mixed with DMEM medium—20 µM, 40 µM, and 80 µM (Table 1)—were freshly prepared and exposed to HaCaT, A549, and HepG2 cells for 24 h. We selected 20 µM of dimethyl-benzanthracene (DMBA) (39570 Merck-Sigma-Aldrich, Budapest, Hungary) dissolved in 1% dimethyl sulfoxide (DMSO) (Merck-Sigma-Aldrich, Budapest, Hungary, catalog number D2650) for positive control. Each experiment was repeated three times (biological replicates) to ensure reproducibility of the results. The medium containing TRZ was aspirated after 24 h, and then to stop the treatment, phosphate buffer saline was used to rinse the cells.

### 2.3. RNA Extraction and Quantitative Real-Time PCR Analysis

Following the manufacturer’s instructions, total RNA was extracted using a semiauto-mated procedure made possible by the Maxwell^®^ RSC Instrument (Promega, Fitchburg, WI, USA) and a Maxwell^®^ RSC RNA FFPE Kit (AS1440, Promega, Fitchburg, WI, USA). Thermo Scientific NanoDropTM 2000 (Thermo Fisher Scientific, Grand Island, NY, USA) was utilized to assess the purity of the RNA. The final step involved dissolving RNA in 50 µL of RNase-free water.

A Roche 480 system was used to perform quantitative real-time PCR tests (Roche, Basel, Switzerland) using the GoTaq^®^ 1-Step RT-qPCR System (Promega GmbH), following the manufacturer’s instructions. The protocol included switch transcribing at 37 °C for 15 min, followed by reverse transcriptase deactivation and hot-start activation at 95 °C for 10 min. Subsequently, 40 cycles were performed, including 10 s of denaturation at 95 °C, 30 s of annealing at 60 °C, and elongation at 72 °C for 30 s. The target genes *DNMT1*, *DNMT3A*, *DNMT3B*, *HDAC5*, and *HDAC6* were furthermore utilized as internal controls in addition to the housekeeping gene hypoxanthine-guanine Phosphoribosyl transferase 1 (*HPRT1*), the detailed forward and reverse primer sequences for the mentioned genes are available in our previous study [35]. The primers and the basic sequences of these genes were created with Integrated DNA Technologies (Bio-Sciences, Budapest, Hungary) and Primer Express TM 3.0.1 Software (Applied Biosystems, Budapest, Hungary). The results were analyzed using the relative quantification technique known as (2^−∆∆CT^) [36].

### 2.4. Assessment of Genomic DNA Stability

Prior to performing the comet assay, cell viability was determined using the trypan blue exclusion method in accordance with the manufacturer’s instructions. Briefly, 10 µL of the cell suspension was mixed with 10 µL of 0.4% trypan blue dye (Merck-Sigma-Aldrich, Budapest, Hungary, catalog number T8154). The mixture was then loaded onto a dual-chamber cell counting slide (Bio-Rad, Budapest, Hungary), and the percentage of viable cells was quantified using the TC20™ automated cell counter (Bio-Rad Inc., Hercules, CA, USA). The viability assay was performed in Duplicate for each sample, and cell viability were above 75%. Using the alkaline comet assay, the effect of various concentrations of tartrazine on genomic DNA integrity was evaluated in HaCat, HepG2, and A549 cells, and for control, the seeded cells received only the medium. A total of 0.5 mL of the cell culture was resuspended in physiological saline. For slide preparation, 150 μL of 0.5% normal melting point agarose (NMA) was spread on each slide and covered with a coverslip to allow solidification. Next, 500 μL of 1% low melting point agarose (LMPA) was mixed with 50 μL of cell culture, and 100 μL of this mixture was layered onto the first agarose coating. A third layer, consisting only of 100 μL 1% LMPA, was then applied on top. After each layer, a coverslip was gently placed to ensure even distribution of the agarose. The slides were incubated in a dark, humid chamber at 37 °C for at least 60 min, followed by immersion in a lysis solution (2.5 M NaCl, 1% sodium sarcosinate, 100 mM Na_2_ -EDTA, 10% DMSO, 10 mM Tris, and 1% Triton X-100) for 1 h at 4 °C. Subsequently, they were placed in an electrophoresis buffer containing 200 mM EDTA and 10 mM NaOH (pH 10) for 20 min. Electrophoresis was then carried out in the dark for 40 min at 132 mA and 0.46 V/cm. After electrophoresis, in 5 min intervals, the slides were neutralized three times with a 0.4 M Tris solution and stained with 50 μL ethidium bromide. At least 50 nuclei per slide were analyzed using Comet Assay IV Imaging Software, Version 4.3.1 (400× magnification; Perceptive Instruments Ltd., Bury St Edmunds, UK), and DNA damage was quantified based on the tail moment parameter evaluated and measured my comet assay IV software (Perceptive Instruments Ltd., Bury St Edmunds, UK).

### 2.5. Statistical Analysis

For the qRT-PCR results, Kolmogorov-Smirnov Test was performed to test normality, the Levene statistic to assess the homogeneity of variance, the one-way ANOVA test to compare means, and the Tukey and Tamhane tests to identify significant differences during the post hoc analysis. Additionally, for the comet assay results, we performed the Mann–Whitney U test to evaluate statistical significance. Statistical analysis was executed using IBM SPSS Statistics for Windows, Version 26.0 (Armonk, NY, USA). Differences were considered significant at *p* < 0.05.

## 3. Results

The research evaluated the expression levels of epigenetic-associated enzymes, specifically *DNMT1*, *DNMT3a*, *DNMT3b*, *HDAC5*, and *HDAC6*, in HaCaT, HepG2, and A549 cell cultures. Relative mRNA expression was measured using *HPRT1* as the reference gene for normalization. We present the results of relative gene expression following exposure to TRZ concentrations ranging from 20 to 80 µM compared to 20 µM DMBA as positive control.

Overall, our findings indicate that TRZ has increased the gene expression of the mentioned genes by 0.5 to 1-fold compared to DMBA, a well-known carcinogen.

### 3.1. TRZ Exposure Significantly Upregulates DNMT1, DNMT3a, and DNMT3b Gene Expression in a Dose-Dependent Manner Across HaCaT, HepG2, and A549 Cell Lines

**Figure 2:**
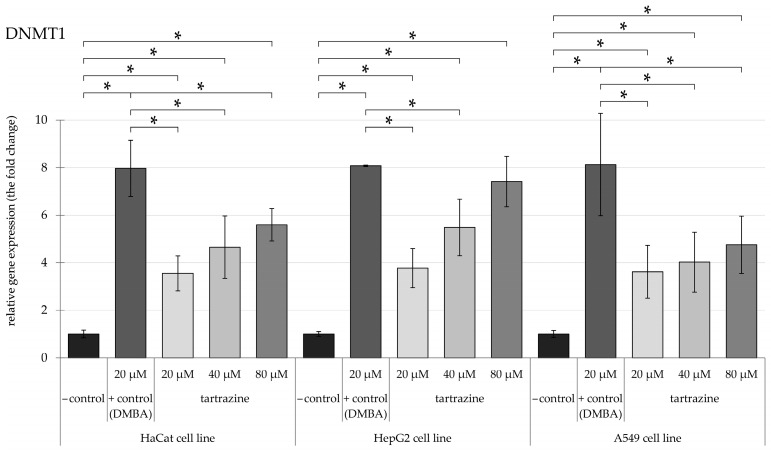
Highlights changes in DNMT1 gene expression in HaCaT, HepG2, and A549 cells, with significance at *p* < 0.05, following exposure to TRZ at concentrations of 20, 40, and 80 µM over 24 h (* = *p* < 0.05).

Concerning *DNMT1* (Figure 2), TRZ significantly elevated the *DNMT1* relative gene expression level compared to the negative control across all cell lines. In HaCaT cells, TRZ at 20 µM increased gene expression significantly compared to the control. There was a significant increase in the relative gene expression level in the HepG2 cell line, with increases of over 4-fold, 6-fold, and notably 8-fold after administering the 20, 40, and 80 µM of TRZ, respectively. However, in the A549 cell line, the level of *DNMT1* exhibited a consistent 4-fold significant increase in all the mentioned concentrations.

**Figure 3:**
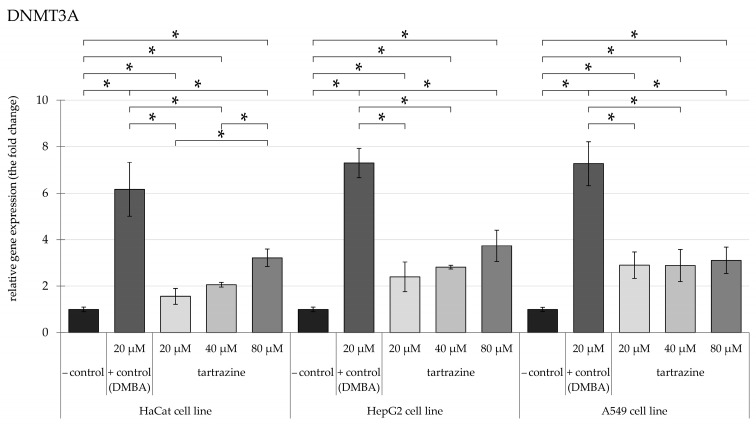
Highlights changes in DNMT3a gene expression in HaCaT, HepG2, and A549 cells, with significance at *p* < 0.05, following exposure to TRZ at concentrations of 20, 40, and 80 µM over 24 h (* = *p* < 0.05).

Derived from the analysis of *DNMT3a* (Figure 3), the findings revealed a dose-dependent, statistically significant elevation in the HaCaT cell line following exposure to varied TRZ doses. When compared to the group under control, there was a 4-fold increase following the administration of 80 µM of TRZ. In the HepG2 cell line, the level of *DNMT3a* gene expression also exhibited a significant dose-dependent elevation, ranging from 2-fold to 4-fold, respectively. The A549 cell line exhibited a significant 3-fold increase relative to the negative control at all selected TRZ concentrations.

**Figure 4:**
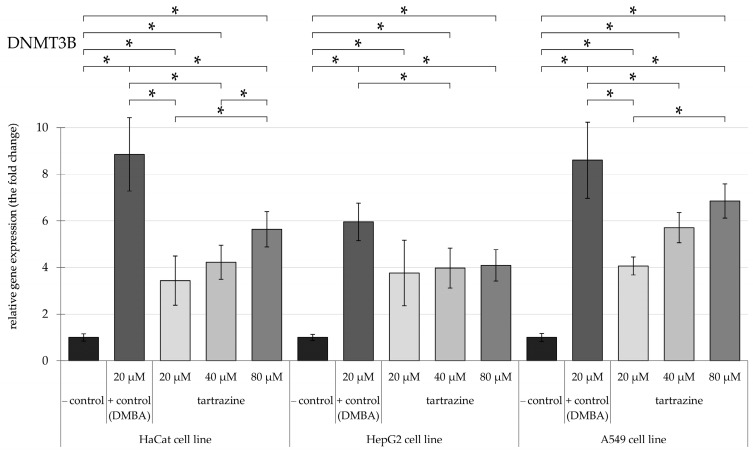
Highlights changes in DNMT3b gene expression in HaCaT, HepG2, and A549 cells, with significance at *p* < 0.05, following exposure to TRZ at concentrations of 20, 40, and 80 µM over 24 h (* = *p* < 0.05).

Exposure to TRZ resulted in an elevated level of *DNMT3b* (Figure 4) across all tested cell lines. In HaCaT cells, the expression of this enzyme gene increased in a dose-dependent manner, reaching an approximately 6-fold rise at 80 µM concentrations. Even the lowest concentration of tartrazine in HepG2 cells quadrupled the mRNA gene expression level compared to the control; however, increasing the dose did not cause any further increase in *DNMT3b* expression levels.

Additionally, in the A549 cell line, the level of *DNMT3b* gene expression nearly reached an almost 8-fold increase at an 80 µM concentration, standing for a 6-fold higher.

### 3.2. TRZ Exposure Induces Significant Overexpression of HDAC5 and HDAC6 Genes in HaCaT, HepG2, and A549 Cell Lines

**Figure 5:**
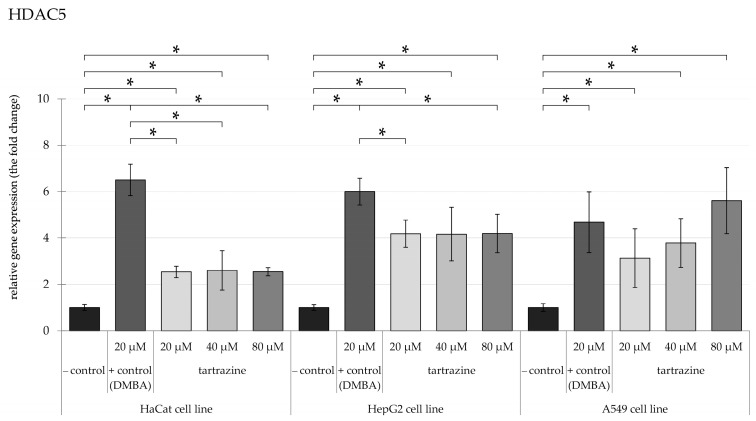
Highlights changes in HDAC5 gene expression in HaCaT, HepG2, and A549 cells, with significance at *p* < 0.05, following exposure to TRZ at concentrations of 20, 40, and 80 µM over 24 h (* = *p* < 0.05).

Expression at 40 µM concentration. Concerning the impact of TRZ on *HDAC5* (Figure 5), gene expression in HaCaT and HepG2 cell lines, a nearly a 3-4-fold significant increase was observed after exposure to 20 µM, reaching a plateau even at the higher concentration. Meanwhile, in A549 cells, the gene expression of *HDAC5* reached an almost 6-fold increase after exposure to 80 µM of TRZ.

**Figure 6:**
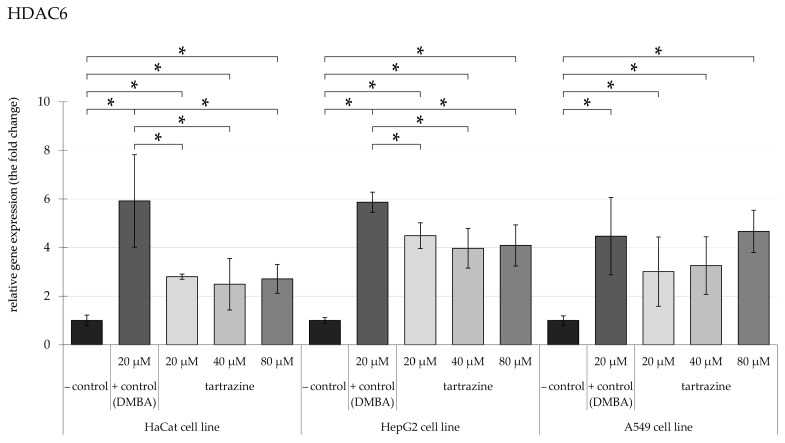
Highlights changes in HDAC6 gene expression in HaCaT, HepG2, and A549 cells, with significance at *p* < 0.05, following exposure to TRZ at concentrations of 20, 40, and 80 µM over 24 h (* = *p* < 0.05).

A comparable outcome was noted in the expression of the *HDAC6* (Figure 6) gene, mirroring the pattern observed for *HDAC5* expression in HaCaT and HepG2 cell lines. There was a significant overexpression of nearly 4-fold. In the A549 cell line, the overexpression was measured fivefold, and after exposure to 20 µM and 40 µM of TRZ, a following less than 1.5-fold significant increase was observed after 80 µM of TRZ.

### 3.3. TRZ Induces Dose-Dependent DNA Damage in HaCaT, A549, and HepG2 Cell Lines

**Figure 7:**
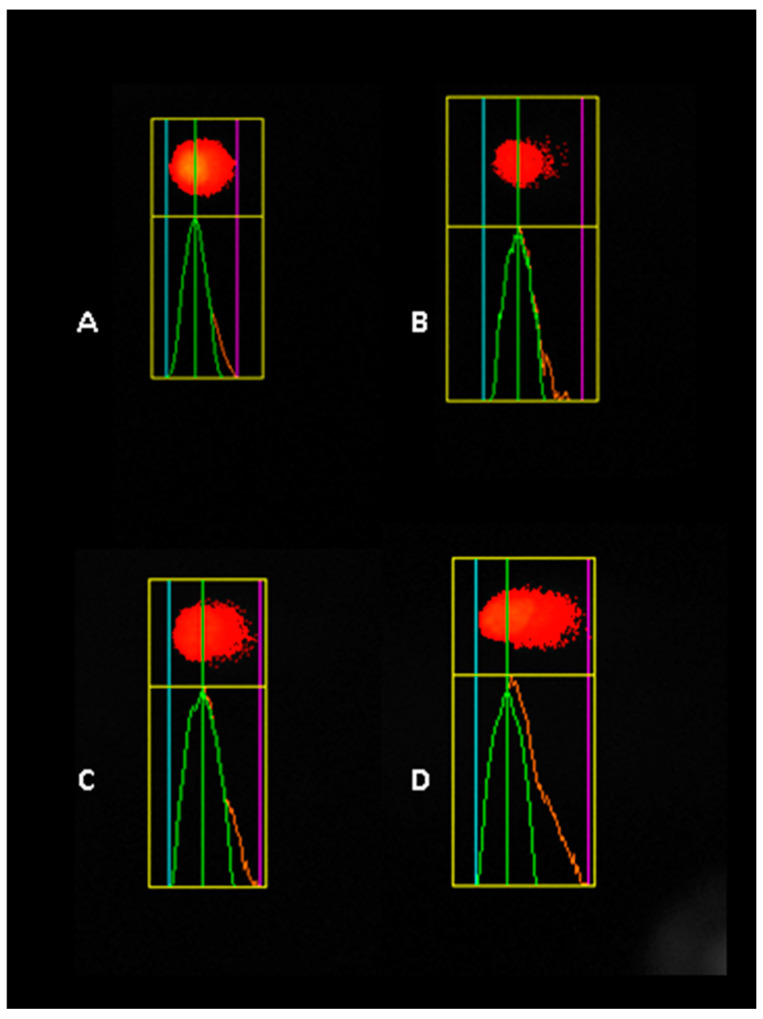
Representative image of the comet assay illustrating DNA damage in HaCaT cells exposed to different concentrations of tartrazine: (**A**) control, (**B**) 20 µM, (**C**) 40 µM, and (**D**) 80 µM.

Our study highlights that Tartrazine exposure significantly increases DNA damage, measured as tail moments, in HaCaT, A549, and HepG2 cell lines, pointing to its potential genotoxic effects. In HaCaT cells, we observed a clear dose-dependent pattern, with DNA damage significantly higher by 2-fold at 40 µM and nearly 3.7-fold at 80 µM compared to both the control group and the 20 µM treatment (Figure 7).

**Figure 8:**
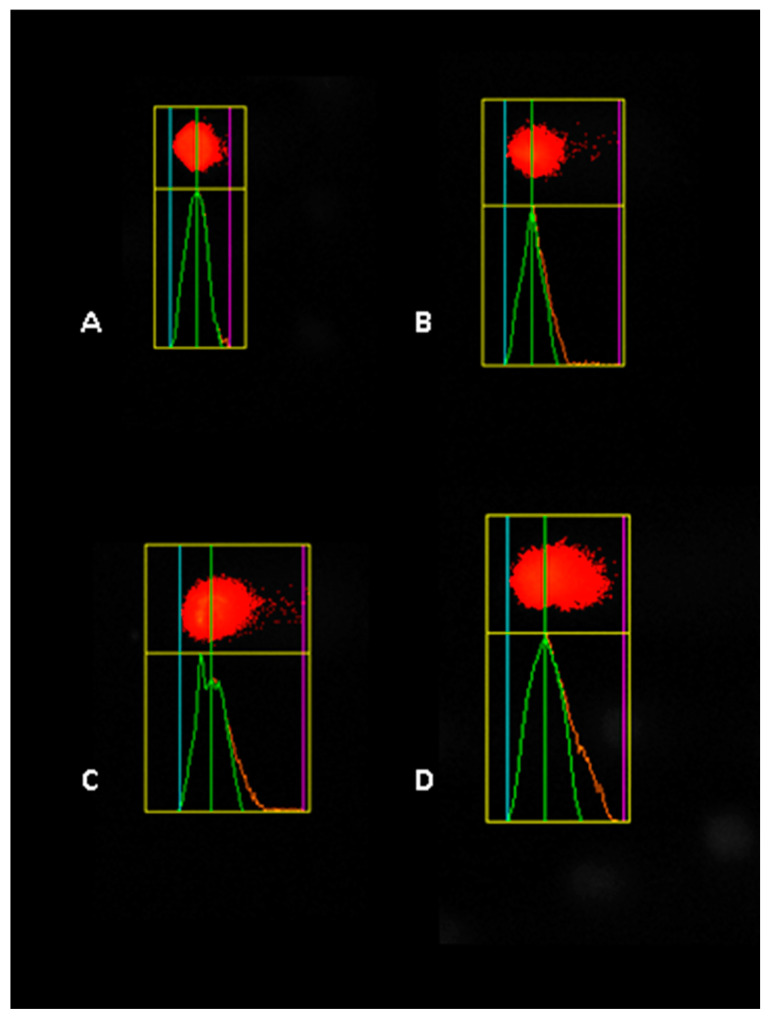
Representative image of the comet assay illustrating DNA damage in A549 cells exposed to different concentrations of tartrazine: (**A**) control, (**B**) 20 µM, (**C**) 40 µM, and (**D**) 80 µM.

In A549 cells, the impact of tartrazine was equally notable (Figure 8), with statistically significant increases in DNA damage across all tested concentrations (* *p* < 0.05). At 20 µM and 40 µM, tail moments nearly doubled compared to the control, while at 80 µM, the damage increased to a nearly 3-fold increase, highlighting the compound’s amplified genotoxic potential at higher doses.

**Figure 9:**
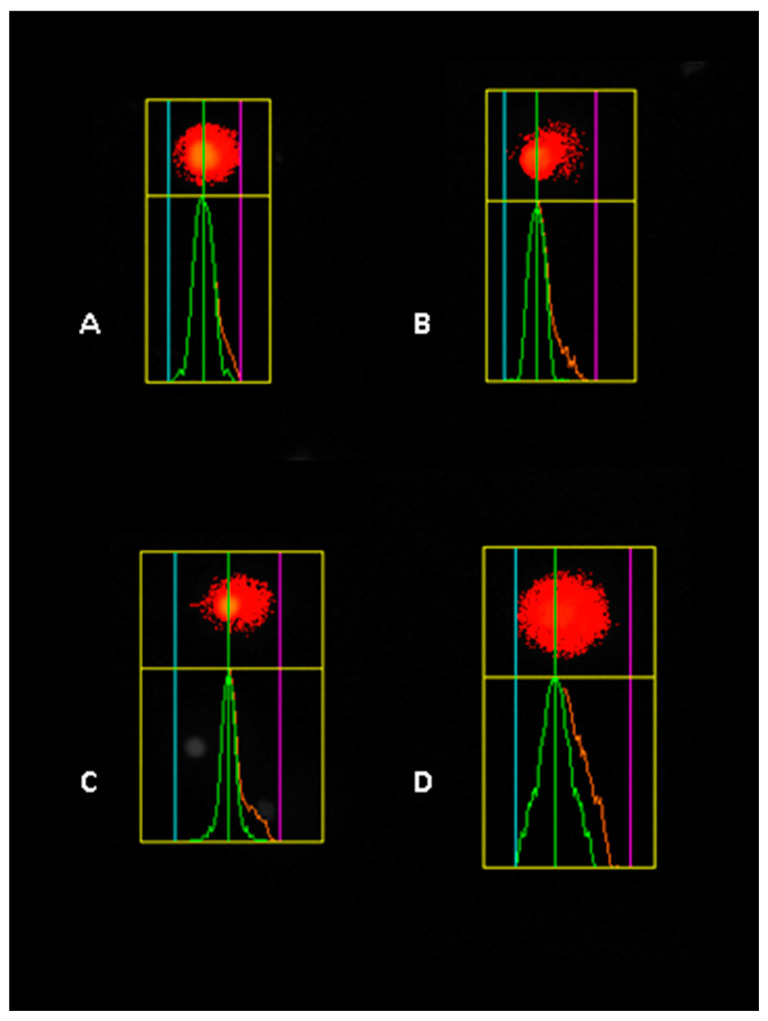
Representative image of the comet assay illustrating DNA damage in HepG2 cells exposed to different concentrations of tartrazine: (**A**) control, (**B**) 20 µM, (**C**) 40 µM, and (**D**) 80 µM.

HepG2 cells also showed a dose-dependent increase in DNA damage (Figure 9), with a nearly two-fold rise observed at 80 µM relative to the control group. However, the extent of DNA damage at 80 µM in HepG2 cells was slightly lower than in HaCaT and A549 cells, suggesting that HepG2 cells may exhibit reduced sensitivity or a distinct response to Tartrazine exposure.

**Figure 10:**
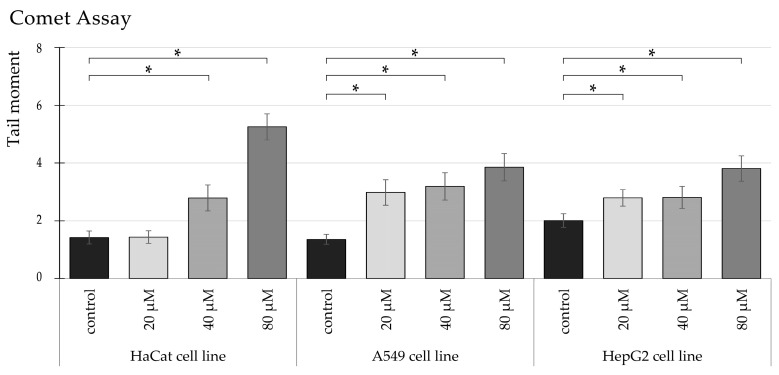
Highlights changes in DNA fragmentation (tail moments) in HaCaT, A549, HepG2 cells treated with TRZ at 20, 40, and 80 µM over 24 h (* = *p* < 0.05).

In summary, our results confirm that tartrazine induces significant DNA damage in a concentration-dependent manner across all three cell lines (Figure 10). The variations in the magnitude of DNA damage suggest cell-line-specific differences, possibly influenced by their unique metabolic or repair mechanisms.

## 4. Discussion

Our research investigated how the presence of TRZ, a food azo dye, affects a specific group of genes involved in DNA epigenetic modification, namely *DNMTs* and *HDACs*, across various human cell lines. DNMTs represent a crucial category of enzymes that regulate epigenetic expression. They are responsible for the regular endogenous alteration of eukaryotic DNA, known as DNA methylation, which is essential to life. This process does not alter the DNA sequence or the gene product. However, it may modify how a gene is expressed [37]. After DNA synthesis, DNMT1s primary role is to keep the methylation state intact. De novo DNA methylation is catalyzed by DNMT3a and DNMT3b, which also catalyzes the methylation of several genomic DNA locations in vivo [38]. Compared to DNMT3a, DNMT3b has more methylation activity on nucleosomal DNA and bare DNA. The core region of nucleosomes contains DNA that is little methylated by DNMT3a, but DNMT3b methylates this DNA considerably, albeit with little activity. Studies have shown that the significant methylation activity of DNMT3b on the DNA in the nucleosome core and the preferential methylation activity of DNMT3a on bare nucleosomal DNA facilitate the remarkable methylation of genomic DNA in vivo [39].

In a recent study, administering TRZ at both low and high levels for a duration of 56 days resulted in elevated levels of alanine aminotransferase (ALT) and aspartate aminotransferase (AST) enzymes in male rats. Prior research has indicated that food coloring agents like TRZ could elevate oxidative stress by promoting the excessive generation of ROS in the liver [40]. In vivo, research has shown that both low and high doses of TRZ result in elevated levels of hepatic serum enzymes specifically ALT and AST, and oxidative stress biomarkers in rodent models [41].

Tartrazine induces oxidative stress in Chinese hamster ovary cells by lowering GSH levels and elevating MDA levels. This may compromise cellular defenses, potentially leading to various diseases connected to oxidative stress, such as cell death [42].

One study by Shakoor and colleagues demonstrates the increased level of reactive oxygen species, as well as glutathione reductase, compared to the control group following exposure of rats to oral TRZ. Research exploring the precise molecular pathways in the lungs impacted by TRZ remains limited. Certain studies have indicated that TRZ might trigger oxidative stress and inflammation [43]. Nevertheless, it could play a role in lung injury and worsen respiratory conditions like asthma. Increased DNMT activity in epithelial cells was discovered to be correlated with higher levels of prostaglandin E2 and cyclooxygenase-2 (Cox-2) by Ram Prasad’s research team [44]. According to this study, TRZ has elevated the relative expression of *HDACs* and *DNMTs* in A549 cells, suggesting a possible negative effect on lung health via inducing inflammation, oxidative stress, and the elevation of pro-inflammatory cytokines.

Recent findings propose that even minor quantities of azo dyes absorbed via tattoos could prompt an immune reaction [45]. Upon treatment of the different cancer cell lines (HaCaT, HepG2, and A549 cells) with TRZ in our study, *DNMT1*, *DNMT3a*, and *DNMT3b* were significantly upregulated at concentrations of 20, 40, and 80 µM for a duration of 24 h. While DNMT1 methylates DNA to maintain methylation patterns during cell division, DNMT3a and DNMT3b are essential for the establishment of de novo DNA methylation [46]. Our study depicts that increased expression of *DNMT 1* in HaCaT, HepG2, and A549 cells is crucial for certain cancer categorization, early detection, therapy, and the forecasting of metastasis and recurrence [47,48,49]. These findings have been supported by Zhang and colleagues, who investigated similar DNA methylation in cervical cancer. Moreover, reports have said that *DNMT1* silencing induces apoptosis and suppresses proliferation [50]. Certain bioactive dietary ingredients can exhibit anti-cancer effects by inhibiting DNA methyltransferase and reducing DNA hypermethylation of key genes. Examples include genistein (soybeans), dietary polyphenols (various plant sources), certain isothiocyanates, and (−)-epigallocatechin-3-gallate (EGCG) from green tea. Their cancer-suppressive activity is partly due to the reactivation of genes, including tumor suppressors, through the demethylation of previously silenced promoters [51]. Dietary polyphenols, such as EGCG, can directly inhibit DNMT1 by interacting with its catalytic site and may also indirectly influence methylation through metabolic pathways. Consequently, dietary DNMT inhibitors hold promise for supplementary cancer treatment and prevention by reversing the hypermethylation-induced silencing of key tumor suppressor genes [51]. Synthetic food colorants like tartrazine have been linked to DNA methylation changes that contribute to genomic instability. In contrast, natural dietary compounds, such as indicaxanthin—a betalain pigment found in prickly pear—have shown beneficial effects by inducing autophagy and facilitating DNA demethylation in colorectal cancer cells. These findings highlight the dual role of dietary components in epigenetic regulation, with their impact varying based on their chemical composition and metabolic interactions, potentially promoting either carcinogenic or protective outcomes [52]. Our findings, therefore, explicitly demonstrate the potential activity of TRZ on critical gene expression common in HaCaT, HepG2, and A549 cells.

Histone deacetylases (HDACs) are chromatin-modifying enzymes that control epigenetic gene expression. Numerous cellular functions depend on HDACs, which deacetylate histone and nonhistone proteins. There are reports of *HDAC* overexpression in various cancer forms associated with faster cell division and survival [53]. *HDAC5* (a class IIa *HDAC*) is one of the eighteen human HDACs that has been identified to be involved in dynamic processes like myoblast differentiation [54], neuronal regrowth and restoration, and synoviocyte activation. Nowadays, research has shown that hepatocellular carcinoma and high-risk medulloblastoma exhibit aberrant overexpression of *HDAC5*, while colon cancer [55] and lung cancer patients’ poor prognosis are linked to *HDAC5* downregulation [56]. In human breast cancer, *HDAC5* stimulates proliferation, invasion, and migration, making it a possible therapeutic target and prognostic indicator [57]. Our findings demonstrated a generalized upregulation of *HDAC5* and *HDAC6* genes in HaCaT, HepG2, and A549 after treatment exposure with TRZ, indicating that *HDAC5* and *HDAC6* are potential lung, liver, and skin cancers therapeutic targets and prognostic biomarkers.

The comet assay is a powerful tool for monitoring human and environmental exposures to genotoxic substances and can aid in hazard characterization for risk assessment [58,59]. In our previous research, we have discussed that, gastrointestinal microflora can metabolize tartrazine into the aromatic amine sulfanilic acid, which may generate reactive oxygen species (ROS) and lead to oxidative stress. In rats, tartrazine induces dose-dependent DNA damage in the colon, potentially leading to tumor formation. Peroxynitrite, formed by the reaction of nitric oxide with ROS, may further contribute to DNA damage [60]. In this study, we observed a significant increase in DNA damage from tartrazine exposure in all studied human cell lines, measured by comet assay. Long-term studies by Himri et al. show that acceptable daily intake (ADI) levels of tartrazine and its byproducts can produce ROS, inducing oxidative stress that affects hepatic and renal structures and their biochemical profiles. Notably, tartrazine exposure also significantly reduces total antioxidant capacity [61]. El-Desoky and colleagues observed a genotoxic effect of tartrazine on bone marrow cells in treated rats, as indicated by the comet assay. Compared to controls, the percentage of DNA in comet tails rose significantly (*p* < 0.01) following tartrazine exposure [62].

Azo dyes like tartrazine are the most commonly utilized colorants in the cosmetics industry. They are found in lipsticks, facial products, toothpaste, mouthwash, shampoos, detergents, and other everyday items. Moreover, they serve as dyes for wool and silk [47]. Human skin can directly absorb azo dye from clothing [48]. Direct contact of the skin with azo dyes such as TRZ could result in allergic responses like urticaria and eczema [49]. Urticaria has been directly associated with abnormal expression of *DNMT*; research indicates that DNA methylation alterations can impact T cells’ multiplication, stimulation, and specialization [50,51]. Another study found that *HDAC* inhibitors can inhibit mast cell activation, which also plays a crucial role in allergic reactions [52]. Elevated expression of *DNMTs* and *HDACs* induced by TRZ may exacerbate conditions like urticaria and eczema. Therefore, we encapsulate the detrimental effects of TRZ applied as a colorant in various human applications, in relation to specific cancer types’ of growth and proliferation.

## 5. Conclusions

This study, to the best of our knowledge, is the first to examine the impact of tartrazine on *DNMTs* as well as *HDACs* genes in human cell lines. Tartrazine significantly upregulates the gene expression of *DNMTs* and *HDACs* in the experimented cell lines, which play a role in cell proliferation. Comparing our data with existing literature, we propose a hypothesis that the consumption of tartrazine might promote the growth of cells and potentially elevate the risk of cancer development. These findings highlight the urgent need for further investigation in this area to better understand the potential health risks associated with tartrazine consumption.

## Figures and Tables

**Table 1:** Tartrazine treatment protocol.

Group ID	Group Name	Food Colorant Concentration (μM)	Description
1	Negative Control (Medium)	0	Baseline control group with no treatment
2	Treated Group 1 (Low Dose)	20	Low-concentration treatment group
3	Treated Group 2 (Medium Dose)	40	Medium concentration treatment group
4	Treated Group 3 (High Dose)	80	High-concentration treatment group
5	Positive Control (DMBA)	-	Control group treated with DMBA as a positive reference
6	Untreated Control (1% DMSO)	-	Control group treated with DMSO as a solvent control

## Data Availability

Data will be made available upon request due to the fact that this manuscript is part of a multiple study.

## Abbreviations

The following abbreviations are used in this manuscript:

ADI	linear dichroism
EFSA	European Food Safety Authority
FDA	Food and Drug Administration
A549	human lung adenocarcinoma
Ach	acetylcholine
ALT	alanine aminotransferase
AST	aspartate aminotransferase
Cox-2	cyclooxygenase-2
DMBA	dimethyl-benzanthracene
DMEM	Dulbecco’s altered Eagle’s medium
DMSO	dimethyl sulfoxide
DNMT	DNA methyltransferases
DNMT1	DNA methyltransferases 1
DNMT3	DNA methyltransferases 3b
DNMT3a	DNA methyltransferases 3a
EGCG	(−)-epigallocatechin-3-gallate
FBS	fetal bovine serum
GABA	gamma-aminobutyric acid
GSH	glutathione
HaCaT	immortalized human keratinocyte
HepG2	human hepatocellular carcinoma
HPRT1	hypoxanthine-guanine Phosphoribosyl transferase 1
MDA	malondialdehyde
NOAEL	No observed adverse effect level
ROS	reactive oxygen species
RT-qPCR	reverse transcription-quantitative polymerase chain reaction
TRZ	tartrazine
TSGs	tumor suppressor gene
